# Reports on the Hospitals of the United Kingdom

**Published:** 1910-11-12

**Authors:** Henry Burdett


### REPORTS
ON THE
### Hospitals of the United Kingdom.

By SIR HENRY BURDETT, K.C.B., K.C.V.O.

### LV.—ESSEX COUNTY HOSPITAL, COLCHESTER.

In 1896 we made a careful inspection of this hospital and recommended certain rather drastic changes in its administration, including a material alteration in the methods upon which the out-patient department was conducted. These recommendations included structural, administrative, and detail changes which the committee accepted at that time. Since then a new children's ward has been erected and a home for the accommodation of the nurses, and other minor but important additions have been made. A study of the reports for the last three years led us to conclude that the committee had succeeded in greatly improving the hospital in all its departments, and this impression was confirmed by a personal inspection of the institution which we made on November 3. The main thing which is lacking to make this institution a vigorous county hospital of the best type, apart from certain structural faults which cannot be changed, is that the people of Colchester, to whose needs in the day of sickness and accident this institution has faithfully ministered for ninety consecutive years, shall at length realise that they have a hospital, and that hospital care is a material asset in the welfare and progress of the town and its inhabitants. Attempts have been made by various speakers at various times, and by influential and earnest townsmen of Colchester, to arouse an interest in the hospital, but to-day it is probably the fact that there is no town containing 40,000 inhabitants which, speaking generally, is so indifferent to and unconscious of the work of the county hospital as the town of Colchester. We wish that every inhabitant of Colchester could realise that for fifty years the Essex County Hospital was the only institution which provided hospital care for the people of Essex, inside and outside Colchester itself.

### The Buildings.

So far as the hospital buildings are concerned, although, with the exception of the new children's ward and the nurses' home, they are not modern and have necessary defects due to the latter fact, most of them may be regarded as fairly satisfactory. The mortuary department is, however, unworthy of a county hospital. There is no mortuary chapel, and, when we inspected the mortuary, there were two bodies in shells at one side, which was screened off by a curtain from the post-mortem table, the condition of which was not creditable to those responsible for its condition. No time should be lost therefore in providing a mortuary chapel apart from the post-mortem room. We were surprised and shocked to see the mortuary of a county hospital as we found this one on the day of our visit. Again, the slip of a room used by the secretary, where three women clerks have sometimes to work, is ill-ventilated and cannot be regarded as healthy. The great cupboards which it contains should be removed into the board-room, together with the secretary's own table, and he should use this room as his office when the Board does not sit. The other room should be ventilated by making a window into the corridor at one end, so as to allow of through ventilation, and proper desk accommodation should be provided for the use of the clerks.

Although the sleeping accommodation for the nurses is on the whole satisfactory, the furniture in the sisters' and nurses' sitting rooms is inadequate and unworthy. More comfortable chairs should be supplied without delay for the use of the staff. The same remark applies to the general room, which is also used as a dining-room for the residents; it has a bare and uncomfortable appearance that is quite unnecessary, and could easily be remedied by spending a few pounds in decoration and the provision of suitable furniture of various kinds. The impression given to the visitor is that no one appears to have realised that those who reside and work habitually in a hospital ought to have comfortable quarters in which they can spend their off-duty hours.

Again, the larder, so called, is not suitable for the purposes to which it is put, owing to its situation and the absence of adequate ventilation. The air in this larder, although the door was kept wide open, was bad. We would suggest that the end room should be converted into a larder and the present larder converted into a room and store for the cook-housekeeper after the ventilation of both rooms has been attended to. These relatively small matters will not cost much money to adjust, and they ought to be put in hand without delay.

### Finance and Economics.

It is satisfactory to note that the Committee have kept a watchful eye on the expenditure, and that this control is continuously present. The report records that the average total cost of each in-patient during the year 1909, compared with that of the previous year, shows a reduction of 10s. per in-patient, representing a saving of approximately £500 for the year. The medical work of the hospital seems to be thoroughly well done, for the average number of days each in-patient was resident was twenty-seven in 1909, showing a reduction of nine days' residence per patient compared with the previous year. County hospitals ought to be hospitals in fact as well as in name, as the medical authorities of Colchester seem from the foregoing figures rightly to recognise.

Turning to the income account it appears that there is an energetic endeavour on the part of the secretary and Finance Committee to keep up the income, and the figures show that annual subscriptions have increased from £1,807 in 1906 to £1,876 in 1909. The donations and boxes too yielded £955 in 1909, compared with £507 in 1906. On the other hand the Hospital Sunday collections are decreasing at a rate which, if it is not stayed, will soon bring this item of income to the vanishing point. Thus Hospital Sunday produced £538 in 1906, but there has been a steady falling-off every year since, and in 1909 it only produced £477. We cannot believe the clergy and ministers of all denominations can contemplate these figures with satisfaction, whether they regard them from the hospital's point of view or their own. Few if any hospitals in this country would, we believe, reveal such a gradual loss of touch with the people by the clergy and ministers of a large town as that which is exhibited by the figures we have just quoted. The Hospital Saturday collections were not satisfactory during 1909, but the whole scheme of collections is in process of reorganisation, and we have little doubt that they will soon produce a revenue of at least £1,000 per annum.

It is but just to the Committee to state that, having regard to the actual cost per patient and per bed under all items of expenditure, it is not possible for the managers to provide adequately for the patients and upkeep of the institution at a less cost than the accounts for 1909 show. We commend these facts to the people of Colchester, who should show their appreciation by coming forward in much greater numbers as subscribers and donors to the hospital.

### Administration and Management.

What we have just said proves that, so far as the committee and secretariat are concerned, the work of the hospital is being zealously and well done. We visited the hospital at 10 o'clock in the morning, and were surprised to find that the wards were at that time being got ready, and that some of them were not in order. We may mention that, for the first time in our experience, we found the nickel table in the operation theatre covered with dust, and that it was possible to write upon it with one's finger. The impression we formed was that there is an absence of organised control and efficient supervision and discipline within the hospital, and these matters demand and should receive prompt attention, so as to secure that the domestic work of the hospital shall be completed, so far as the wards, operation theatre, and other offices are concerned, at a much earlier hour in the morning than appears to be the case at present. We noticed a few flock pillows in some of the wards, and we hope that these will be replaced by horsehair bolsters and feather pillows. The reports published by the Local Government Board prove that flock ought never to be used for bedding in a well-administered hospital. We observed too that it is desirable that more blankets should be supplied to the tuberculous patients who sleep in the open air. The sisters and nurses were evidently interested in their work, but one sister and one probationer are not a sufficient number to have charge of 23 beds. It is just to add that, so far as our observations went, we formed the conclusions that the patients were comfortable and doing well, although no doubt the comfort of everybody would be increased if a greater knowledge and a closer attention to details in connection with the internal work of the hospital were secured. The hospital needs someone to be constantly in the wards who has a thorough knowledge of hospital management and an apprehension of the many details of such management which combine to secure efficiency throughout a hospital in the daily working. Having regard to the condition of the larder, the wards and the operation theatre, and generally, we are of opinion that steps should be taken to amend the present system so as to make the discipline and orderly arrangement of everything inside the hospital more perfect than it is at present.

### The Hospital's Needs and How to Meet Them.

At the commencement of 1910 the hospital was £4,000 in debt. We understand that this sum has now been provided and the debt written off. The hospital requires a further £1,000 to be expended upon the mortuary, furniture, and other small matters to which we have called attention. The great need of the hospital is, however, more income, and to make the whole administration as efficient and perfect as possible £2,000 additional revenue should be provided by those who use the hospital. Colchester has a population of upwards of forty thousand, and that portion of the county which sends patients to this institution contains at least four hundred thousand inhabitants. £2,000 a year is not a great sum to ask this large population of nearly half a million people to provide without delay.

Having gone closely into the accounts and present financial condition of the hospital we are of opinion that this extra revenue may be readily raised from the following sources. Through the Hospital Saturday and Workshop collections an additional income of at least £400 a year. Mrs. Egerton Green has started a work guild, and this should be joined by the ladies of Colchester and the neighbourhood in sufficient numbers to secure that this guild takes over the whole supply of linen for the hospital, and presents the hospital each year with all it requires in this respect to a value of £100. Next, the villages and urban communities throughout each district from which patients are sent should be treated as individual units; a statement should be got out showing the number of patients sent from each of these centres; the cost entailed by the treatment of these patients and the actual money given by the inhabitants of each, so as to show whether the subscriptions bear any adequate proportion to the actual cost of treating the patients admitted to the hospital. We believe that if this were done an additional income of £500 per annum could easily be raised. The remaining £1,000 per annum of income should be obtained in Colchester itself by the organisation of a hospital association which every member of the community should be asked to join who has any adequate sense of the gratitude they owe to the Great Giver of all good for the blessings of health. Each member should be asked to raise at least 5s. a year in income from small subscribers of 1s. a year each. If this step be taken not only will the additional revenue be speedily forthcoming, but in a few years the inhabitants of Colchester will recognise that the establishment of such an association has had a material influence for good upon all classes and especially upon those individuals who have shown their appreciation of the privilege of rendering personal service in the days of health in the cause of the sick by becoming members of the Colchester Hospital Association. We hope we have made it clear that the task of raising the additional £2,000 a year, provided only the people of Colchester and the district can be aroused to a sense of their responsibilities and privileges, should not only prove easy of accomplishment, but of great benefit to the healthy as well as to the sick.

